# Phytochemical residue profiles in rice grains fumigated with essential oils for the control of rice weevil

**DOI:** 10.1371/journal.pone.0186020

**Published:** 2017-10-12

**Authors:** S. Ezhil Vendan, S. Manivannan, Anila M. Sunny, R. Murugesan

**Affiliations:** Food Protectants and Infestation Control Department, CSIR-Central Food Technological Research Institute, Mysore, India; National Taiwan University, TAIWAN

## Abstract

In the present study, we investigated the fumigant potential of five edible essential oils (EOs) against *Sitophilus oryzae* and their phytochemical residues in treated grains. Among the tested EOs, peppermint oil proved significantly effective (P ≤ 0.05) on *S*.*oryzae* at 400 μl/L air concentration, inducing 83 and 100% mortalities in with-food and without-food conditions respectively over 72 h exposure. In addition, it was also observed that the binary mixtures of peppermint + lemon oil (1:1 ratio) produced an equivalent effect to that of peppermint oil alone treatments. The phytochemical residue analysis by GC-MS revealed the presence of six compounds upon 72 h exposure to EOs. Further, the analysis of physico-chemical properties of the compounds indicated a positive correlation between polar surface area (PSA) and its residual nature. The residue levels of eugenol were significantly elevated corresponding to its high PSA value (29) in clove and cinnamon oils. On the other hand, the compounds with zero PSA value imparted very less or no (D-Limonene, caryophyllene, pinene and terpinolene) residues in treated grains. With respect to the most active peppermint oil, L-menthone, menthyl acetate and eucalyptol residues were at 67, 41 and 23% levels respectively. The outcome of the present study indicate the peppermint oil as a potent fumigant against *S*. *oryzae*, and although the residues of phytochemicals in treated grains is higher; they belong to the generally recognised as safe (GRAS) status leaving no harmful effect.

## Introduction

Plant essential oils (EOs) are considered as alternatives to conventional pesticides and have been well documented for their toxicity on stored product insect pests. Nearly, 3000 EOs has been reported from plants and about 10% of oils are commercially used as flavour and fragrants [1]. Naturally, EOs are derived from different parts of plant tissues, which play a vital role in plant defense system [2–3]. The EOs comprises of complex mixture of phytochemicals belonging to multiple groups and is responsible for various bioactivities in target insect pests. Among the phytochemicals in EOs, terpenes are known to have high vapour pressure and can easily diffuse in air at normal atmosphere [4]. Monoterpenes (C_10_H_16_) are volatile in nature and are responsible for fumigant actions in EOs [5]. Many studies have demonstrated the fumigant action of monoterpenes (e.g., limonene, menthone, eugenol, eucalyptol and menthol) against a range of insect pests [6–9].

Most recently, Isman and Grieneisen [10] stated that insecticide research on essential oils has increased nearly 15% during the past decade. However, no information is available in the literature regarding the commercialization of EO based fumigants for stored product insect control. In India, phytochemical based pesticides for commercial use are very less and EO based fumigants are nil as per Central Insecticides Board and Registration Committee [11]. Thus, alternative strategies and adequate data are required to formulate EO based fumigants. Rajendran and Sriranjini [12] had suggested that, further studies are required to understand the phytochemical residue/sorption levels on different food commodities and in EOs mixtures to validate the potentiality of EO based fumigants for stored product insect pest control. However, very less reports are available currently on the phytochemical residual analysis in EO fumigated commodities considering their high molecular weight and low vapour pressure. Similarly, the data on commercial use of EOs as food preservative agents remains unclear in the context of phytochemical residues and their toxicities [13].

The rice weevil, *Sitophilus oryzae* (L.) is one of the most destructive and cosmopolitan insect pest of stored cereals worldwide. Presently, phosphine is predominantly used across the globe to eradicate this pest species effectively from the infested commodities [14]. Due to resistance development in insects, the target concentration of phosphine required to achieve 100% insect control has increased from 80 ppm to 1000 ppm during the years 1992 to 2001 [15]. Reddy et al. [16] stated that, complete mortality of *Sitophilus* spp. population can be achieved at a dosage of 1 g/m^3^ phosphine held for seven days. Consequently, it is also known that, *S*. *oryzae* is one of the most tolerant species and had rapidly developed strong resistance to phosphine [17]. A wide range of plant based EOs have been screened for insecticidal activity targeting different modes of action on different insect species. However, the reports pertaining to phytochemical residue analysis on fumigated commodities still remains less. Therefore, the present investigation was undertaken to validate the fumigant potential of five edible EOs *viz*., Cinnamon (*Cinnamomum zeylanicum*), Clove (*Syzygium aromaticum*), Lemon (*Citrus limon* L.), Orange (*Citrus aurantium*) and Peppermint (*Mentha piperita*) against *S*. *oryzae* and to determine their phytochemical residues in treated rice grains. The fumigant toxicity of EOs on *S*. *oryzae* was compared in with and without-food conditions and the residual levels of active ingredients were also analysed. In addition, composition and physico-chemical properties of phytochemicals have been correlated with their residual property and accordingly discussed in this article.

## Materials and methods

### Solvents, chemicals, essential oils and other raw materials

HPLC grade solvents, EOs of five selected plants *viz*., cinnamon, clove, lemon, orange and peppermint and phytochemicals (eucalyptol, neoisocarvomenthol, menthone and menthyl acetate) were procured from Sigma-Aldrich Chemicals Ltd, India. Aluminium phosphide tablets ‘Quickphos’ (United Phosphorus Ltd., Mumbai, India) was obtained from commercial pesticide supplier. Rice grains (Raw rice, MR-gold variety) used for the experiment was procured from Local Super Market, Mysore, Karnataka, India. The obtained rice grains were held at -20°C in deep freezer (Blue Star, HF 300 model) for a week to disinfest any field carried infestation. The typical moisture content of the rice grains used for the experiment was about 12%.

### Insect culture

Insect cultures of rice weevil, *S*. *oryzae* was regularly maintained in the insectary unit (with controlled condition 30±2°C, 75±5% humidity and 13:11—light:dark photoperiod) in Food Protectants and Infestation Control department at CSIR-Central Food Technological Research Institute, Mysore. A group (approximately 1000 individuals) of *S*. *oryzae* adults obtained from the stock culture were introduced into 2 L capacity individual glass jars containing 1 Kg rice grains. The insect culture was under regular observation until enough adults were developed. From this sub culture, adults of similar age groups were obtained and used for fumigant toxicity studies.

### Fumigant bioassays

Individual EOs, phytochemicals and binary mixtures of EOs were performed in fumigant bioassays. Glass tubes (28 x 114 mm; 50 ml capacity) fitted with glass stoppers were used for performing the fumigation bioassays. Whatmann No. 1 filter paper strips (20 mm square) were prepared and pasted to the lower side of stopper. The top layer of each tube (about one inch length from the filter paper) was lined with Insect-A-slip to avoid insects coming in direct contact with the filter paper strips. All EOs, phytochemicals and binary mixtures were evaluated for fumigant toxicity both in with and without-food conditions. For fumigant bioassays under with-food conditions, 5 gm of rice was added to each tube. Ten individual adults of 15 days old were released into each tube. Among them, three highly active EOs were chosen to study the effect of binary mixtures of EO combinations in 1:1 ratio. Based on the individual EO fumigant bioassay results, major phytocompounds from the most active EO was selected for further studies. Different concentrations *viz*., 20, 100, 200, 400 μl/L air of EOs (including binary mixtures i.e., peppermint+lemon, peppermint+orange and lemon+orange) and phytocompound samples were loaded onto the filter paper. After loading each test concentrations, the open end of the individual tube was held with stoppers and further sealed with thin polythene films to make it air tight. Phosphine gas was used as a positive control for the phytocompound fumigant bioassay. Phosphine gas generation and fumigant dosing at various concentrations (20, 100, 200, 400 μl/L air) was carried out by following Manivannan et al., method [14]. Three sets were maintained for each of the treatment to observe mortalities at 24, 48 and 72 h exposures respectively. Likewise, the entire experiment was repeated in five replications.

### Phytochemical residue extraction

After 72 h exposure to respective EOs, the treated rice samples were extracted from the tubes and were subjected to phytochemical residue analysis. The fumigated rice grains obtained from all the five replicates were pooled together for phytochemical residue analysis. Accordingly, twenty-five grams of rice in each concentration was soaked in methanol at 1:3 (rice: methanol; w/v) ratio. The residual extract of each sample was collected and filtered through Whatmann No. 1 filter paper. Likewise, the extraction procedure was repeated thrice for each test sample. Obtained residual extracts were concentrated using a rotary vacuum evaporator. The final concentrates of the phytochemical extracts were stored in deep freezer (-20°C) for further GC-MS analysis.

### GC-MS analysis

The GC-MS analysis was performed on a Perkinelmer system equipped with Turbo mass Gold mass spectrometer (Norwalk, CT. USA). Elite-5 capillary column (30 m x 0.25 mm, 0.25 μm film thickness) was used for analysis. Helium served as the carrier gas and all the samples were analyzed by the following specifications; Initial temp 40°C for 2 min, ramp 5°C/min to 290°C, Inj = 250°C, Volume = 1 μL, Split = 10:1, Delay: 5.00 min, Transfer Temp = 200°C, Source Temp = 180°C, Scan: 29 to 400Da. Compounds were identified by comparison of their respective mass spectra, retention indices (Kovats index) and above 40% of relative abundance of acceptance match criteria with those of standards and by comparing with the NIST mass spectral data system/library.

### Data processing and statistical analysis

After the fumigant bioassays were complete, the per cent corrected mortalities were estimated following Abbot’s [18] formula for each of the treatment and represented along with their mean and standard error; percentage corrected mortality = (percentage mortality in treatment—percentage mortality in control/100—percentage mortality in control) x 100. One-way analysis of variance (ANOVA) with Tukey’s multiple range tests were performed for the mortality data to determine the significant difference between treatments and to identify the effective treatment at P ≤ 0.05 for further binary mixture studies. Lethal concentration for 50% mortality (LC_50_) was estimated for 72 h exposure for all the EOs and binary mixtures by probit analysis [19]. ANOVA and probit analysis were performed using SPSS statistical software (version 16.0). From the GC-MS data, relative concentration (RLC) and residue concentration (RSC) of each component in percentage was calculated by following formulas: RLC = (Component area / Total area) x 100; RSC = [(Sample area x Volume of sample) / (Reference area x Volume of reference)] x 100.

## Results

### Response of *S*. *oryzae* to EO treatments in without-food conditions

The results indicated that all the tested EOs in without-food condition exhibited concentration vs time dependent activities on *S*. *oryzae* (Table 1). After 24 h exposure, no or remarkable mortality was observed in any of the treatments, except for orange and peppermint oils which recorded 40 and 34 per cent mortalities at 400 μl/L air concentration. A minimum test concentration of 100 μl/L air was required to achieve more than 10% of mortality in *S*. *oryzae* for all the EO treatments exposed for 48 h. Among the five tested EOs, peppermint, lemon and orange oils displayed significant potential activities (>95 per cent mortality) at 400 μl/L air concentration exposed to *S*. *oryzae* over 72 h exposure (P ≤ 0.05). Complete adult mortality was recorded in peppermint and lemon oils treatments at maximum test concentration and long exposures.

**Table 1:** Mortality of *S*. *oryzae* adults exposed to five edible essential oils at different concentrations and times under without-food conditions.

Essential oils	Concentration (μl/L air)			
	20	100	200	400
**24 h treatment**				
Cinnamon	0.00 ± 0.00^a^	0.00 ± 0.00^a^	0.00 ± 0.00^a^	0.00 ± 0.00^a^
Clove	0.00 ± 0.00^a^	0.00 ± 0.00^a^	0.00 ± 0.00^a^	0.00 ± 0.00^a^
Lemon	0.00 ± 0.00^a^	0.00 ± 0.00^a^	0.00 ± 0.00^a^	6.00 ± 5.98^a^
Orange	0.00 ± 0.00^a^	0.00 ± 0.00^a^	6.00 ± 3.99^a^	40.00 ± 5.47^b^
Peppermint	0.00 ± 0.00^a^	0.00 ± 0.00^a^	0.00 ± 0.00^a^	34.00 ± 6.77^b^
**48 h treatment**				
Cinnamon	4.08 ± 2.49^a^	8.16 ± 5.58^a^	10.20 ± 4.99^a^	12.24 ± 6.91^a^
Clove	0.00 ± 0.00^a^	10.20 ± 3.81^a^	14.29 ± 5.19^ab^	24.49 ± 6.11^a^
Lemon	0.00 ± 0.00^a^	20.41 ± 3.81^ab^	57.14 ± 3.81^c^	89.79 ± 6.44^c^
Orange	2.04 ± 2.49^a^	10.20 ± 4.99^a^	28.57 ± 4.56^b^	65.31 ± 5.19^b^
Peppermint	0.00 ± 0.00^a^	36.73 ± 4.99^b^	100.00 ± 0.00^d^	100.00 ± 0.00^c^
**72 h treatment**				
Cinnamon	16.33 ± 4.99^ab^	22.45 ± 2.49^a^	24.49 ± 2.49^a^	36.73 ± 3.81^a^
Clove	0.00 ± 0.00^a^	18.37 ± 3.22^a^	22.45 ± 2.49^a^	44.90 ± 6.91^a^
Lemon	32.65 ± 2.49^b^	46.94 ± 6.76^ab^	61.22 ± 5.94^b^	100.00 ± 0.00^b^
Orange	51.02 ± 3.41^c^	59.18 ± 3.22^b^	71.43 ± 3.81^b^	95.92 ± 4.07^b^
Peppermint	12.24 ± 6.11^a^	77.55 ± 14.55^b^	100.00 ± 0.00^c^	100.00 ± 0.00^b^

Each value is a mean of five replicates with standard error (Mean ± SE). Means within a column and within exposure period (h) followed by the different letters are significantly different (P≤0.05) of mortality of *S*. *oryzae* adults as determined by Tukey’s test.

### Response of *S*. *oryzae* to EOs treatments in with-food conditions

Mortality of the test insects decreased in fumigant bioassays carried out under with-food conditions (Table 2) than without-food (Table 1). Similar to without-food assay, adult mortality was also dependent on the concentration and exposure. However, lemon and orange oils exhibited moderate activities (55 and 46 respectively) at higher concentration and longer exposure. Maximum mortality (83%) was observed in peppermint oil treatment at 400 μl/L air concentration over 72 h exposure. Among the tested EOs, peppermint oil showed significant activities at P ≤ 0.05 at the test concentrations from 100 to 400 μl/L air over 72 h exposure.

**Table 2:** Mortality of *S*. *oryzae* adults exposed to five edible essential oils at different concentrations and times under with-food conditions.

Essential oils	Concentration (μl/L air)			
	20	100	200	400
**24 h treatment**				
Cinnamon	0.00 ± 0.00^a^	0.00 ± 0.00^a^	0.00 ± 0.00^a^	4.00 ± 2.45^a^
Clove	0.00 ± 0.00^a^	0.00 ± 0.00^a^	0.00 ± 0.00^a^	0.00 ± 0.00^a^
Lemon	0.00 ± 0.00^a^	0.00 ± 0.00^a^	0.00 ± 0.00^a^	2.00 ± 1.99^a^
Orange	0.00 ± 0.00^a^	0.00 ± 0.00^a^	0.00 ± 0.00^a^	0.00 ± 0.00^a^
Peppermint	0.00 ± 0.00^a^	0.00 ± 0.00^a^	2.00 ± 1.99^a^	6.00 ± 2.45^a^
**48 h treatment**				
Cinnamon	2.04 ± 2.49^a^	4.08 ± 2.49^a^	10.20 ± 3.81^a^	16.33 ± 3.81^a^
Clove	0.00 ± 0.00^a^	8.16 ± 4.56^ab^	12.24 ± 4.07^a^	16.33 ± 2.04^a^
Lemon	0.00 ± 0.00^a^	10.20 ± 3.81^ab^	20.41 ± 5.94^a^	30.61 ± 3.81^ab^
Orange	0.00 ± 0.00^a^	4.08 ± 2.49^a^	18.37 ± 3.22^a^	20.41 ± 3.81^a^
Peppermint	16.33 ± 3.81^b^	20.41 ± 3.81^b^	26.53 ± 2.04^a^	36.73 ± 4.99^b^
**72 h treatment**				
Cinnamon	10.20 ± 3.81^a^	18.37 ± 3.22^a^	20.41 ± 5.94^a^	32.65 ± 4.07^ab^
Clove	8.16 ± 6.44^a^	20.41 ± 3.81^a^	24.49 ± 2.49^ab^	26.53 ± 2.04^a^
Lemon	28.57 ± 3.22^bc^	42.86 ± 4.07^b^	44.89 ± 2.49^c^	55.10 ± 5.19^c^
Orange	16.33 ± 3.81^ab^	24.49 ± 2.49^a^	38.78 ± 3.22^bc^	46.94 ± 3.81^bc^
Peppermint	38.78 ± 3.22^c^	59.18 ± 4.56^c^	65.31 ± 4.07^d^	83.67 ± 2.49^d^

Each value is a mean of five replicates with standard error (Mean ± SE). Means within a column and within exposure period (h) followed by the different letters are significantly different (P≤0.05) of mortality of *S*. *oryzae* adults as determined by Tukey’s test.

### Fumigant toxicities of phytocompounds of most active peppermint oil on *S*. *oryzae*

The fumigant toxicities of selected four phytocompounds on *S*. *oryzae* are depicted in Table 3. Complete mortality (100%) of *S*. *oryzae* was observed in eucalyptol and menthone treatments at 200 and 400 μl/L air concentrations over 72 h exposure under without-food conditions. In with-food condition, more than 80% mortality was recorded for eucalyptol and menthone treatments at the highest test concentrations and maximum exposure. When compared to phosphine treatment (positive control), eucalyptol and menthone showed significant activities at P ≤ 0.05 in without and with-food conditions for 400 μl/L air concentration at 72 h exposure. Followed by eucalyptol and menthone, menthyl acetate also exhibited fumigant activities. No significant mortality was noticed for neoisocarvomenthol treatments over 24 h exposure, either in without-food or with-food condition.

**Table 3:** Mortality of *S*. *oryzae* adults exposed to selected four phytocompounds at different concentrations and times under with and without-food conditions.

Condition	Compounds	Concentration (μl/L air)			
		20	100	200	400
*Without-food*	**24 h treatment**				
	Eucalyptol	0.00 ± 0.00^a^	9.09 ± 6.38^a^	87.88 ± 5.88^c^	95.96 ± 4.03^d^
	N. Menthol*	0.00 ± 0.00^a^	5.05 ± 2.47^a^	9.09 ± 4.51^a^	11.11 ± 5.88^ab^
	Menthone	0.00 ± 0.00^a^	1.01 ± 2.02^a^	3.03 ± 4.03^a^	7.07 ± 3.77^a^
	M. Acetate^#^	15.15 ± 6.04^b^	21.21 ± 8.67^a^	39.39 ± 5.52^b^	47.47 ± 4.94^c^
	Phosphine	0.00 ± 0.00^a^	6.00 ± 2.45^a^	12.00 ± 3.74^a^	32.00 ± 3.16^bc^
	**48 h treatment**				
	Eucalyptol	0.00 ± 0.00^a^	53.54 ± 5.14^d^	100.0 ± 0.00^c^	100.0 ± 0.00^c^
	N. Menthol*	7.07 ± 5.88^ab^	13.13 ± 2.47^a^	23.23 ± 5.14^a^	27.27 ± 3.77^a^
	Menthone	0.00 ± 0.00^a^	37.37 ± 5.88^bc^	71.72 ± 7.41^b^	95.96 ± 2.47^c^
	M. Acetate^#^	19.19 ± 4.51^b^	23.23 ± 4.03^ab^	59.60 ± 3.19^b^	61.62 ± 11.67^b^
	Phosphine	8.00 ± 5.82^ab^	32.00 ± 3.74^ab^	52.00 ± 5.82^b^	66.00 ± 3.99^b^
	**72 h treatment**				
	Eucalyptol	10.20 ± 9.88^a^	69.39 ± 3.22^b^	100.0 ± 0.00^c^	100.0 ± 0.00^b^
	N. Menthol*	14.29 ± 8.28^a^	32.65 ± 6.91^a^	42.86 ± 2.49^a^	51.02 ± 4.99^a^
	Menthone	28.57 ± 7.20^a^	65.31 ± 5.19^b^	100.0 ± 0.00^c^	100.0 ± 0.00^b^
	M. Acetate^#^	36.73 ± 7.48^a^	51.02 ± 5.94^ab^	69.39 ± 4.56^b^	87.76 ± 4.99^b^
	Phosphine	13.13 ± 2.47^a^	65.66 ± 5.14^b^	73.74 ± 2.47^b^	85.86 ± 4.03^b^
*With-food*	**24 h treatment**				
	Eucalyptol	0.00 ± 0.00^a^	0.00 ± 0.00^a^	9.09 ± 5.52^a^	89.89 ± 7.81^c^
	N. Menthol*	0.00 ± 0.00^a^	0.00 ± 0.00^a^	0.00 ± 0.00^a^	0.00 ± 0.00^a^
	Menthone	0.00 ± 0.00^a^	0.00 ± 0.00^a^	0.00 ± 0.00^a^	0.00 ± 0.00^a^
	M. Acetate^#^	0.00 ± 0.00^a^	0.00 ± 0.00^a^	0.00 ± 0.00^a^	0.00 ± 0.00^a^
	Phosphine	2.00 ± 2.00^a^	8.00 ± 3.74^a^	34.00 ± 5.09^a^	42.00 ± 5.82^b^
	**48 h treatment**				
	Eucalyptol	0.00 ± 0.00^a^	5.05 ± 4.03^a^	31.31 ± 5.88^c^	97.98 ± 2.02^d^
	N. Menthol*	0.00 ± 0.00^a^	0.00 ± 0.00^a^	0.00 ± 0.00^a^	0.00 ± 0.00^a^
	Menthone	0.00 ± 0.00^a^	11.11 ± 3.77^a^	55.56 ± 2.47^d^	73.74 ± 4.03^c^
	M. Acetate^#^	3.30 ± 2.49^a^	7.07 ± 2.02^a^	15.15 ± 4.03^b^	21.21 ± 3.77^b^
	Phosphine	6.00 ± 2.45^a^	40.00 ± 4.46^b^	48.00 ± 3.74^d^	62.00 ± 5.82^c^
	**72 h treatment**				
	Eucalyptol	6.12 ± 3.81^a^	24.49 ± 9.45^b^	87.75±4.99^cd^	100.0 ± 0.00^c^
	N. Menthol*	0.00 ± 0.00^a^	0.00 ± 0.00 ^a^	0.00 ± 0.00^a^	0.00 ± 0.00^a^
	Menthone	14.29 ± 5.19^a^	42.86 ± 6.91^bc^	100.0 ± 0.00^d^	100.0 ± 0.00^c^
	M. Acetate^#^	8.16 ± 3.22^a^	28.57 ± 1.77^b^	38.78 ± 4.56^b^	42.86 ± 8.28^b^
	Phosphine	16.00 ± 5.09^a^	68.00 ± 6.62^c^	80.00 ± 4.46^c^	88.00 ± 2.00^c^

*Neoisocarvomenthol;

^#^Menthyl acetate;

Each value is a mean of five replicates with standard error (Mean ± SE). Means within a column and within exposure period (h) followed by the different letters are significantly different (P≤0.05) of mortality of *S*. *oryzae* adults as determined by Tukey’s test.

### Response of *S*. *oryzae* to binary mixtures of EOs

The results of the binary mixtures of EOs from lemon, orange and peppermint oils tested against the adults of *S*. *oryzae* are presented in Table 4. All the tested binary mixtures of EOs expressed mortalities following 24 h exposure. Insect mortalities decreased in with-food assay compared to the without-food assay. Peppermint+lemon oil mixture displayed promising activities compared to other binary mixtures exposed for 72 h. The obtained mortalities over exposure to the test concentration of 400 μl/L air for 48 and 72 h exposure was significantly superior (P ≤ 0.05) over the other binary mixtures, resulting in 95 to 100% mortality.

**Table 4:** Mortality of *S*. *oryzae* adults exposed to binary mixtures of edible essential oils at different concentrations and times.

Condition	Essential oils*(1:1)	Concentration (μl/L air)			
		20	100	200	400
*Without-food*	**24 h treatment**				
	Oe+Ln	0.00 ± 0.00^a^	0.00 ± 0.00^a^	0.00 ± 0.00^a^	2.00 ± 1.99^a^
	Pt+Oe	0.00 ± 0.00^a^	0.00 ± 0.00^a^	0.00 ± 0.00^a^	0.00 ± 0.00^a^
	Pt+Ln	0.00 ± 0.00^a^	0.00 ± 0.00^a^	12.00 ± 5.82^a^	30.00 ± 4.46^b^
	**48 h treatment**				
	Oe+Ln	0.00 ± 0.00^a^	12.24 ± 2.49^a^	28.57 ± 3.22^a^	38.78 ± 3.22^a^
	Pt+Oe	2.04 ± 2.49^ab^	12.24 ± 4.07^a^	38.78 ± 3.22^a^	51.02 ± 3.81^a^
	Pt+Ln	14.29 ± 5.19^b^	32.65 ± 2.49^b^	36.73 ± 5.94^a^	95.92 ± 4.07^b^
	**72 h treatment**				
	Oe+Ln	6.12 ± 3.81^a^	20.41 ± 2.04^a^	30.61 ± 2.04^a^	53.06 ± 2.49^a^
	Pt+Oe	10.20 ± 3.81^a^	24.49 ± 2.49^a^	40.82 ± 3.81^a^	61.22 ± 5.94^a^
	Pt+Ln	18.37 ± 3.22^a^	36.73 ± 3.81^b^	69.39 ± 4.56^b^	100.00 ± 0.00^b^
*With-food*	**24 h treatment**				
	Oe+Ln	0.00 ± 0.00^a^	0.00 ± 0.00^a^	0.00 ± 0.00^a^	0.00 ± 0.00^a^
	Pt+Oe	0.00 ± 0.00^a^	0.00 ± 0.00^a^	0.00 ± 0.00^a^	0.00 ± 0.00^a^
	Pt+Ln	0.00 ± 0.00^a^	0.00 ± 0.00^a^	0.00 ± 0.00^a^	0.00 ± 0.00^a^
	**48 h treatment**				
	Oe+Ln	2.04 ± 2.49^a^	12.24 ± 2.49^a^	20.41 ± 3.81^a^	26.53 ± 3.81^a^
	Pt+Oe	10.20 ± 3.81^a^	28.57 ± 3.22^b^	40.82 ± 3.81^b^	51.02 ± 3.81^b^
	Pt+Ln	26.53 ± 2.04^b^	30.61 ± 2.04^b^	36.73 ± 3.81^b^	67.35 ± 2.04^c^
	**72 h treatment**				
	Oe+Ln	10.20 ± 2.04^a^	22.45 ± 2.49^a^	28.57 ± 3.22^a^	53.06 ± 5.19^a^
	Pt+Oe	22.45 ± 2.49^b^	34.69 ± 2.49^a^	55.10 ± 2.49^b^	61.22 ± 3.81^a^
	Pt+Ln	48.98 ± 3.22^c^	63.27 ± 5.19^b^	69.39 ± 3.22^c^	85.71 ± 5.19^b^

*Oe—Orange oil; Ln—Lemon oil; Pt—Peppermint oil.

Each value is a mean of five replicates with standard error (Mean ± SE). Means within a column and within exposure period (h) followed by the different letters are significantly different (P≤0.05) of mortality of *S*. *oryzae* adults as determined by Tukey’s test.

The estimated probit analysis data for *S*. *oryzae* exposed to individual EOs, phytocompounds and binary mixtures of EOs for 72 h exposure are shown in Table 5. The LC_50_ concentrations recorded for orange and lemon oils were less than 65 and 550 μl/L air concentration for without-food and with-food exposures respectively. Peppermint oil proved to be the potent EO fumigant, recording the least LC_50_ (47.8 μl/L air) and LC_90_ (124.4 μl/L air) values for without-food exposure compared to the other EOs. Similarly, the peppermint oil treatment also exhibited promising toxicity on *S*. *oryzae* under with-food conditions, recording least LC_50_ (45.2 μl/L air) and LC_90_ (1372.8 μl/L air) values. Moreover, the estimated fiducial limits for LC_50_ and LC_90_ concentrations_,_ cinnamon and clove oils showed wide ranges with maximum lethal concentration values. On the other hand, the most active peppermint oil showed narrow range of fiducial limits; 32.0–66.4 and 87.2–219.6 μl/L air ranges obtained for without-food exposure and 28.0–63.4 and 732.0–3993.4 μl/L air ranges obtained for with-food exposure (LC_50_ and LC_90_ respectively). Compared to the positive control (phosphine), the phytocompounds eucalyptol and menthone showed notable LC_50_ and LC_90_ values than neoisocarvomenthol and menthyl acetate in fumigation bioassays. The LC_50_ and LC_90_ values were <55 and <150 μl/L air in without-food condition and <110 and <300 μl/L air in without-food condition for eucalyptol and menthone respectively. In the EO binary mixture bioassays, the minimal LC_50_ and LC_90_ values were noticed in peppermint+lemon oil mixture. Less than 100 μl/L air values of LC_50_ and LC_90_ values of 427.8 and 1425.4 μl/L air were obtained for peppermint+lemon oil combination in without-food and with-food exposures respectively. Narrow range of fiducial limits was obtained for peppermint+lemon oil combination than the other treatments.

**Table 5:** Probit analysis of mortality for *S*. *oryzae* adults exposed to individual essential oils, phytocompounds and binary mixtures of essential oils for 72 h exposure.

Essential oils*	LC_50_ (μl/L)	95% Confidence Limit (LL–UL)	LC_90_ (μl/L)	95% Confidence Limit (LL–UL)	Slope ± SE	X^2^ (df)
***Oils—Without-food***						
Cn	3356.2	878.6–554252.0	2950159.6	64497.8–1.391E13	0.44 ± 0.07	66.43 (18)
Ce	504.0	353.0–930.2	4169.2	183480.0–20693.4	1.39 ± 0.09	101.87 (18)
Ln	63.0	34.8–96.4	598.6	318.6–2219.8	1.31 ± 0.07	255.48 (18)
Oe	26.8	8.8–46.6	655.4	322.8–3163.6	0.92 ± 0.06	153.64 (18)
Pt	47.8	32.0–66.4	124.4	87.2–219.6	3.08 ± 0.11	398.59 (18)
***Oils—With-food***						
Cn	3066.2	843.2–469073.8	609992.8	24003.4–3.670E11	0.56 ± 0.07	96.87 (18)
Ce	3707.4	856.8–7012794.0	895645.8	23497.0–3.527E14	0.54 ± 0.07	112.17 (18)
Ln	233.4	145.0–511.8	92794.8	12083.8–14224132.8	0.49 ± 0.06	50.52 (18)
Oe	534.6	330.8–1249.8	38814.8	8851.2–750989.2	0.69 ± 0.07	53.31 (18)
Pt	45.2	28.0–63.4	1372.8	732.0–3993.4	0.86 ± 0.06	62.95 (18)
***Phytocompounds—Without-food***						
El	54.2	39.2–71.2	143.0	106.0–222.6	3.04 ± 0.11	285.82 (18)
Nm	327.8	187.2–1084.8	12522.4	2541.0–1437557.0	0.81 ± 0.07	162.52 (18)
Me	40.6	28.4–54.0	154.6	112.2–246.4	2.21 ± 0.09	203.38 (18)
Ma	52.8	27.0–81.6	928.2	451.0–4238.2	1.03 ± 0.06	167.26 (18)
Pe	75.6	59.5–93.1	449.9	333.2–681.6	1.66 ± 0.07	87.82 (18)
***Phytocompounds—With-food***						
El	101.0	68.4–139.6	283.0	195.8–556.8	2.87 ± 0.11	460.47 (18)
Nm	-	-	-	-	-	-
Me	65.8	45.6–89.4	207.2	145.6–362.4	2.57 ± 0.09	336.27 (18)
Ma	472.8	292.4–1167.2	13782.8	3692.8–252515.0	0.88 ± 0.07	96.19 (18)
Pe	66.2	49.5–84.3	370.4	268.7–586.1	1.71 ± 0.07	121.17 (18)
***Oil Mixtures—Without-food***						
Oe+Ln	406.8	297.4–648.2	5211.0	2385.0–19707.4	1.16 ± 0.08	69.04 (18)
Pt+Oe	270.6	199.8–412.6	3533.8	1647.8–13543.0	1.15 ± 0.07	90.24 (18)
Pt+Ln	85.6	61.8–114.2	427.8	286.2–819.0	1.84 ± 0.07	204.59 (18)
***Oil Mixtures—With-food***						
Oe+Ln	463.6	315.8–857.4	10448.2	3760.4–67018.4	0.95 ± 0.07	66.23 (18)
Pt+Oe	173.4	133.8–234.4	6396.2	2867.4–23189.2	0.82 ± 0.06	40.72 (18)
Pt+Ln	24.6	8.0–43.2	1425.4	604.8–9509.8	0.73 ± 0.06	93.03 (18)

*Cn—Cinnamon; Ce—Clove; Oe—Orange oil; Ln—Lemon oil; Pt—Peppermint oil; El—Eucalyptol; Nm—Neoisocarvomenthol; Me—Menthone; Ma—Menthyl acetate; Pe—Phosphine

### Phytochemical residue of EO compounds correlating with the polar surface area

**Fig 1:**
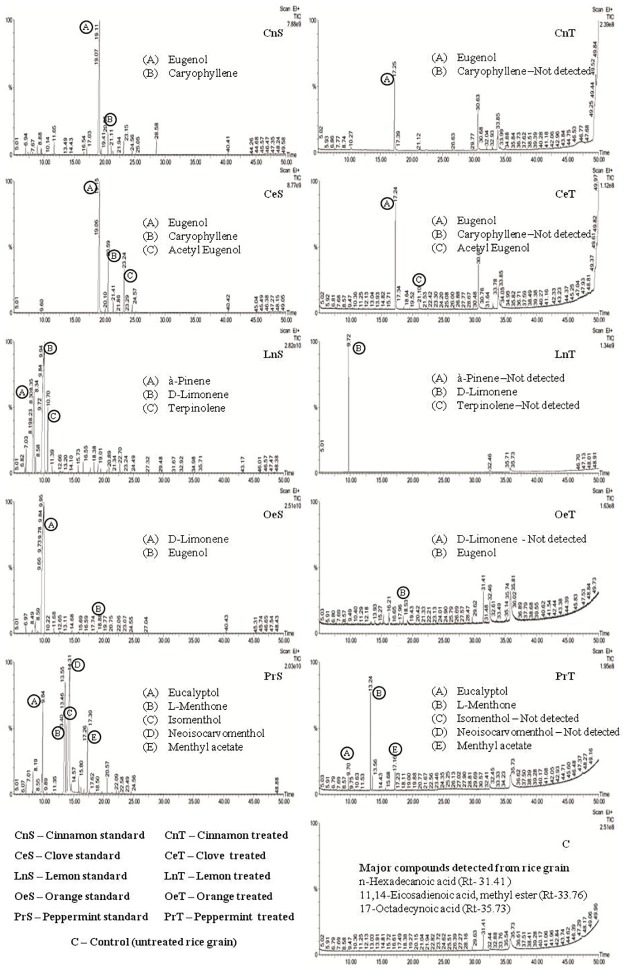
Comparison of GC-MS chromatogram of methanolic extract of fumigated rice grains with standard essential oils.

Phytochemical composition and RLC of compounds obtained from the tested EOs are presented in Table 6. GC-MS analysis revealed that, cinnamon, clove, orange, lemon and peppermint oils contained a total of 27, 17, 25, 23 and 25 compounds respectively. The following are the major compounds identified in the selected EOs; eugenol detected in cinnamon (76.76%) and clove (74.55%) oils, D-limonene in orange (93.48%) oil, D-limonene and α-pinene in lemon (66.60 and 17.30% respectively) oil, and neoisocarvomenthol, 1-menthone and eucalyptol in peppermint (40.55, 27.62 and 10.61% respectively) oil. The phytochemicals were classified into five categories based on the RLC levels (I = 0–5%, II = 6–20%, III = 21–45%, IV = 46–70%, V = 71–100%) in EOs for comparative analysis and presented in Table 7. No RLC value is depicted in the Table for the EO with level I, except for eugenol in orange oil which showed reasonable traces of RSC in the treated samples. The results suggest that the RSC values did not correspond to their respective RLC levels recorded in the study. Over all, about six compounds were only detected as residues in the fumigated rice grains. Menthone, menthyl acetate and eucalyptol residues were detected in peppermint oil treatments. While, eugenol residue was detected in cinnamon, clove and orange oil treatments and D-limonene and acetyl eugenol in lemon and clove oil treatments respectively. Menthone showed maximum RSC value (67.28%) followed by menthyl acetate, eucalyptol, eugenol, acetyl eugenol and D-limonene with 41.77, 23.50, 18.86 and 1.57% RSC’s respectively. While comparing the RSC of a compound with its total composition in the EO, eugenol showed maximum RSC (about 15% in clove and 20% in cinnamon oils). Fig 1 shows GC MS chromatogram of residual compounds detected in rice grains fumigated with peppermint oil. Physico-chemical properties of phytochemicals of EOs were compared with two conventional fumigants, phosphine and methyl bromide (S1 Table). RSC level was the least or nil for the compounds having zero polar surface area (PSA) and no remarkable variation was observed between the RSC and physico-chemical properties of the compounds except PSA.

**Table 6:** Relative concentrations of phytochemicals estimated from five edible essential oils.

RT	Compound name	Relative concentration (%)*				
		Cn	Ce	Ln	Oe	Pr
5.01	1,2-Propanediamine	-	0.03	-	-	-
5.06	Hydrazinecarboxamide	0.28	0.41	-	0.11	-
6.75	β-Thujene	0.10	-	-	0.38	-
6.77	Amphetamine-3-methyl	-	0.05	-	-	-
6.93	à-Pinene	1.53	-	17.30	0.82	1.75
6.99	Acetohydrazide	-	0.04	-	-	-
7.33	Camphene	0.38	-	0.06	-	-
7.46	Borane carbonyl	-	0.03	-	-	-
7.67	Benzaldehyde	0.20	-	-	-	-
8.01	á-Phellandrene	1.14	-	-	0.53	0.11
8.49	á-Myrcene	0.13	-	1.86	2.48	0.07
8.81	Octanal	-	-	-	0.27	-
8.93	2,6-Octadien-1-ol, 2,7-dimethyl-	-	-	0.07	-	-
9.02	3-Carene	0.08	-	-	0.20	-
9.22	Terpinolene	0.22	-	9.43	-	-
9.46	o-Cymene	0.98	-	0.12	-	0.12
9.58	Cyclohexene,1-methyl-5-(1-methylethenyl)-,(R)	0.79	-	-	-	-
9.65	D-Limonene	-	0.06	66.60	93.48	1.70
9.84	Eucalyptol	0.13	-	-	-	10.61
10.27	á-Ocimene	-	-	0.09	-	-
10.49	ç-Terpinene	-	-	-	-	0.07
11.64	Linalool	2.50	-	-	0.38	-
11.77	Butanoic acid, 2-methyl-, 2-methylbutyl ester	-	-	-	-	0.04
11.79	Nonanal	-	-	0.09	0.03	-
12.65	Limonene oxide, cis-	-	-	0.10	0.13	-
12.77	Limonene oxide, trans-	-	-	0.08	0.08	-
13.00	1-Methoxy-1,3-cyclohexadiene	-	-	0.05	-	
13.19	Citronellal	-	-	0.04	0.05	-
13.46	L-Menthone	-	-	-	-	27.62
13.78	Isomenthol	-	-	-	-	6.31
14.30	Neoisocarvomenthol	-	-	-	-	40.55
14.45	trans-p-Mentha-2,8-dienol	-	-	-	0.02	-
14.57	à-Terpineol	-	-	-	-	0.53
14.67	Decanal	-	-	-	0.22	-
15.12	Pentanoic acid, 4-methyl-, 1-buten-1-yl ester	-	-	-	-	0.06
15.79	Pulegone	-	-	-	-	0.88
15.80	Carvone	-	-	-	0.09	0.08
15.82	Cyclopentane,1-methyl-2-acetyl-3-(1-methylethenyl)-	-	-	0.07	-	-
16.18	Piperitone	-	-	-	-	0.33
16.54	Cinnamaldehyde, (E)-	0.87	-	-	-	-
16.55	Citral	-	-	1.11	0.05	-
17.03	trans-Isosafrole	1.05	-	-	-	-
17.26	Menthyl acetate	-	-	-	-	7.45
17.75	3,7-Nonadien-2-one, 4,8-dimethyl-	-	-	0.96	-	-
19.26	2-Ethylbutyric acid, 3-phenylpropyl ester	0.08	-	-	-	-
18.34	Geranyl methyl ether	-	-	-	0.02	-
18.88	Eugenol	76.76	74.55	0.08	0.26	0.07
19.00	Neryl Acetate	-	-	0.49	-	-
19.41	α-Copaene	0.72	-	-	-	-
19.41	à-Cubebene	-	0.35	-	-	-
19.49	Geranyl acetate	-	-	0.22	-	-
19.64	(-)-á-Bourbonene	-	-	-	-	0.10
19.73	Germacrene D	-	-	-	0.04	-
20.10	Methyleugenol	-	0.54	-	-	-
20.12	Dodecanal	-	-	-	0.03	-
20.52	Caryophyllene	3.58	9.44	0.20	0.04	1.02
20.77	1,6-Cyclodecadiene,1-methyl-5-methylene-8- (1-methylethyl)-, [S-(E,E)]-	-	-	-	-	0.05
20.89	Alpha-Bergamotene	-	-	0.36	-	-
21.11	Acetic acid, cinnamyl ester	1.40	-	-	-	-
21.34	cis-á-Farnesene	-	0.01	0.04	-	0.13
21.39	Humulene	0.59	1.56	-	-	-
21.86	cis-muurola-3,5-diene	-	0.01	-	-	-
22.36	Valencen	-	-	-	0.06	-
22.52	(-)-Mintlactone	-	-	-	-	0.22
22.69	á-Bisabolene	-	-	0.58	-	-
23.07	d-Cadinene	0.19	-	-	-	0.06
23.14	Acetyl eugenol	2.20	11.28	-	0.03	-
23.81	Caryophyllene oxide	0.62	1.23	-	-	0.07
25.16	Epoxy (1,11) humulene	0.08	0.12	-	-	-
28.57	Benzyl Benzoate	3.02	-	-	-	-
40.41	Piperonyl butoxide	0.38	0.29	-	0.20	-

RT-Retention time,

*Cn—Cinnamon oil; Ce—Clove oil; Oe—Orange oil; Ln—Lemon oil; Pt—Peppermint oil.

**Table 7:** Phytochemical residues detected in essential oils fumigated rice grains.

Compound name	Name of essential oil	RLC level*	RSC obtained / compound	RSC obtained / oil
Acetyl eugenol	Clove	II	12.16 ± 3.51	1.37 ± 0.39
Caryophyllene	Clove	II	nd	nd
Eucalyptol	Peppermint	II	23.50 ± 4.87	0.25 ± 0.05
Eugenol	Cinnamon	V	17.37 ± 5.67	20.08 ± 7.99
	Clove	V	18.86 ± 4.04	14.06 ± 3.01
	Orange	I	13.43 ± 4.34	0.04 ± 0.01
Isomenthol	Peppermint	II	nd	nd
D-Limonene	Lemon	IV	1.57 ± 0.19	0.10 ± 0.01
	Orange	V	nd	nd
Neoisocarvomenthol	Peppermint	III	nd	nd
L-Menthone	Peppermint	III	67.28 ± 8.79	1.86 ± 0.24
Menthyl acetate	Peppermint	II	41.77 ± 5.14	0.31 ± 0.04
à-Pinene	Lemon	II	nd	nd
Terpinolene	Lemon	II	nd	nd

*I = 0–5%, II = 6–20%, III = 21–45%, IV = 46–70%, V = 71–95%;

Each value is a mean of three replicates with standard error (Mean ± SE).

## Discussion

In spite of many investigations to explore the potential of bio-fumigants for stored product insect control, the major disadvantage is the low vapor pressure and high boiling point leading to absorption/ settling of residues on the treated commodities imparting off odors. Hence, in the present study, we comparatively investigated the fumigant toxic potentialities and the residue profiles of cinnamon, clove, lemon, orange and peppermint EOs in fumigated rice grains. Insect mortality results revealed that, all the tested EOs exerted concentration dependent toxic effects on *S*. *oryzae*. Peppermint oil was regarded as highly toxic; while lemon and orange oils were reported to be moderately toxic to *S*. *oryzae*. Previously, Khani et al. [20] and Shaaya et al. [21] reported 85 and 7.5 μl/L air as the LC_50_ concentrations for peppermint oil treatments against *S*. *oryzae*. Whereas in the present study, LC_50_ values of 47.8 (LCL:UCL = 32.0–66.4) and 45.2 μl/L air (LCL:UCL = 28.0–63.4) were recorded for peppermint oil in both with and without-food conditions. The LC_90_ values for peppermint oil increased in with-food conditions (124.4 to 1372 μl/L air) compared to without-food conditions as similar to the other oils. The obtained slope values significantly decreased (P ≤ 0.05) in with-food conditions for all oils except for the less active cinnamon oil. Hence, it was predicted that fumigant toxicities of EOs decreased under with-food conditions indicating that a proportion of active ingredients/phytochemicals might have got absorbed/adhered onto the rice grains thereby reducing the lethality. Similarly, Rajendran and Sriranjini [22] stated that the toxicity of bio-fumigants against insect pests will be less in with-food conditions due to low vapour pressure (≤1 mmHg) and high sorption by the grains.

Based on the toxicity data of the individual oils, peppermint, orange and lemon oils were selected for the binary mixture fumigant bioassays on *S*. *oryzae*. Mortality results showed that, *S*. *oryzae* was more susceptible to peppermint with lemon oil mixture at P ≤ 0.05. This mixture expressed 100% mortality at 400 μl/L air concentration in without-food condition, which decreased to 85% mortality in with-food condition exposed for 72 h. It was observed that peppermint+ lemon oil mixture (1:1) had more fumigant action than the other two mixtures. Earlier bioactivity studies have indicated that the mixtures of essential oils are more effective than individual oils. For example, Krishnarajah et al. [23] reported that, binary mixtures of essential oils were more toxic to *Sitotroga cerealella* than the individual oils. This also holds good for conventional fumigants like phosphine, which showed synergistic effects on *Rhyzopertha dominica* when combined with CO_2_, and the report suggested that PH_3_+CO_2_ admixture increased the insect mortality than phosphine alone treatments [24]. While in the present study, fumigant toxicity of peppermint oil alone was relatively par to peppermint+lemon oil mixture. Similarly, Nattudurai et al. [25] observed that eucalyptus+camphor oil mixture had similar effects (LC_50_) on *Tribolium castaneum* as posed by the individual oils. In the present study, similar to the results of individual oils, the slope values for the binary mixtures greatly varied between with and without-food conditions. This strongly indicated that the toxicity of EOs is altered by the sorptive nature of the commodity.

Monoterpenoids in EOs are known to play a vital role in mitigating fumigant actions on stored product insect pests [12]. Consequently in the present study, peppermint oil showed promising activities due to the presence of major monoterpenoids such as eucalyptol and menthone. When compared to the commercial fumigant (phosphine), menthone and eucalyptol alone treatments showed significant activities at P ≤ 0.05 and were identified as active compounds in peppermint oil exhibiting fumigant activities. Likewise, orange and lemon oils offered moderate toxicities on *S*. *oryzae* attributed by the presence of D-limonene and α-pinene monoterpenoid compounds. Previously, Lee et al. [26] suggested that menthone had more fumigant potential followed by α-pinene and limonene. Later, eucalyptol was also recognised as potent fumigant against *S*. *oryzae*, *T*. *castaneum* and *R*. *dominica* as described by Lee et al. [6]. The present study also insists the fact that, not all monoterpenoids found in EOs possess fumigant actions. Though, eugenol belongs to monoterpenoid group, it displayed less activity against *S*. *oryzae*, despite having a RLC value around 75% in cinnamon and clove oils. Similarly, Liska et al. [7] also reported that eugenol was not effective as a fumigant against *T*. *castaneum*.

It is imperative that, a thorough knowledge on residual properties of EOs is essential to formulate a bio-fumigant. The present phytochemical residual analysis revealed that, only six compounds settle/adhere on the rice grains out of the total detected compounds in individual EO. Among the tested five EOs, phytochemical residues of eugenol (14–20% of RSC per oil) was remarkably higher in clove and cinnamon oils than the other oils. Further, it was also observed that RSC of phytochemicals were associated with their respective PSAs. It is known that PSA is the electrostatic potential of a substance/compound to form physical interactions between surfaces. Palm et al. [27] stated that PSA has been used for prediction of absorption of drugs in pharmaceutical industry. Accordingly, in the present study, we propose a positive correlation between the PSA values of phytocompounds from EOs with their residual nature. For instance, the residues of major compounds like acetyl eugenol, eucalyptol, eugenol, L-menthone and menthyl acetate were more and correlated with their respective PSA values. The phytochemical analysis of eugenol showed notable RLC and corresponding RSC levels in cinnamon and clove oils. While for orange oil, though the RLC level was very low for eugenol, remarkable RSC was noticed due to its high PSA value (29). Similarly, the RLC values were less for acetyl eugenol and menthyl acetate compounds, whereas the RSC values were more, mainly attributed by its high PSA values (36 and 26 respectively). Contrastingly it was observed that, though D-limonene was a major compound in orange (93%) and lemon (66%) oils, the obtained RSC was very less (0.10% of RSC per oil) in lemon and was not detected in orange oil, due to its zero PSA value. Similarly, the PSA value for the other non-residual compounds such as caryophyllene, pinene and terpinolene is zero though their RLC values were about 6–20% in the respective oils. More interestingly, PSA value and RLC level of L-menthone was less in peppermint oil when compared to eugenol in cinnamon and clove oils though the detected residues were very high (40–70%). In contrast, though PSA and RLC values were at noticeable levels for isomenthol and neoisocarvomenthol compounds they did not impart any residue in the fumigated rice grains. These facts denote the instability of the compounds and probable chemical reactions taking place during fumigations. To support this view, Belitz et al. [28], stated that terpene hydrocarbons undergo rapid autooxidation at atmospheric air in many of the essential oils. Hence it was assumed/predicted that menthol isomers (isomenthol and neoisocarvomenthol) might have autooxidized to L-menthone leading to more residue formation. In addition, it is known that isomenthol and neoisocarvomenthol were not stable when compared to menthol because of an axial methyl group and may rapidly get oxidized to menthone. Interestingly, in the present study it was noticed that insect mortality was nil for neoisocarvomenthol in with-food condition and about 50% mortality (at 400 μl/L air concentration and 72 h exposure) was observed in without-food condition. Probably, it was assumed that, in without-food condition, direct exposure/contact of oxidized neoisocarvomenthol (i.e., menthone) on insects might have induced mortality. On the other hand, in with-food condition, a proportion of oxidized neoisocarvomenthol might have settled on rice grains and the actual concentration required to induce insect mortality may be lacking. Krishnaswamy [29] described the oxidation mechanism of menthol and their isomers into menthone isomers. Furthermore, Pecar and Gorsek [30] had described the reaction kinetics of menthol getting oxidized to menthone under laboratory conditions. According to Lee et al. [6], residual level of phytocompound on the commodity is dependent on the quantity of compound being absorbed. In addition, Cartalade and Vernhet [31] stated that, maximum adsorption of phytochemical was associated to the PSA of the material. Similarly, in the present study, the compounds such as acetyl eugenol, eugenol, menthone and menthyl acetate have more PSA values subsequently imparting higher levels of residues in treated grains. Hence, it is proposed that the PSA value may determine the residual property of compounds getting adhered to the surface of commodity.

Furthermore, Reddy et al. [16] stated that sorption/residue of fumigants on commodities is mainly dependent on the moisture content of the grain, size of particle, composition of fumigant agent, exposure phase and dose. Likewise, the conventional fumigants, phosphine and methyl bromide have zero PSA value thereby the residue formation in fumigated commodities will be very less [22, 32].

## Conclusions

Our study signifies that peppermint oil is a potent bio/phyto-fumigant and could be used for the control of *S*. *oryzae* under storage conditions. In addition, the study implies that peppermint oil can also be used in mixed form with lemon oil for effective fumigations. With respect to the phytochemical residues, the identified major residual compounds such as 1-menthone, menthyl acteate, eucalyptal and eugenol, and their respective oils are generally recognised as safe (GRAS) [33–36]. Accordingly, we study suggests that, peppermint and lemon oils can be considered as safer alternatives for commodity treatments. Also, the phytochemical residues could be probably removed by 24 hours aeration, as suggested by Lee et al. [6] for eucalyptol compound. Likely, the residual compounds in peppermint oil fumigated grains may also be removed upon aeration. In addition, Isman [37] reviewed that residues from edible EOs are beneficial to human health by pass through diet. Furthermore, peppermint oil is exempted from Federal Insecticide, Fungicide, and Rodenticide Act (FIFRA) for pesticide formulation in alone or in combination with other ingredients [38]. Hence, our study suggests peppermint oil as a potent bio-fumigant and could be applied with the reduced residual risk under direct exposure to commodities in alone or in mixture with lemon oil for the control of *S*. *oryzae* in stored product commodities.

## Supporting information

S1 Table Comparative analysis of physico-chemical properties of phytochemicals with conventional fumigants. (DOC)
