# Supplementary material for: Phytochemical residue profiles in rice grains fumigated with essential oils for the control of rice weevil
Source: PLoS One. 2017 Oct 12;12(10):e0186020. doi: 10.1371/journal.pone.0186020 (PMC5638326; doi:10.1371/journal.pone.0186020)
Supplement: S1 Table — (DOC) [file pone.0186020.s001.doc]

| **Compound name** | **Molecular weight (g/mol)** | **Density (g/cm^3^)** | **Vapour pressure (mmHg at 25°C)** | **Enthalpy of vaporization (KJ/mol)** | **Polar surface area (**Å^2^**)** |
| --- | --- | --- | --- | --- | --- |
| ***Phytochemicals of EO fumigants*** | | | | | |
| Acetyl eugenol | 206.23 | 1.1 | 0.0 ± 0.5 | 50.6 ± 3.0 | 36 |
| Caryophyllene | 204.35 | 0.9 | 0.0 ± 0.3 | 48.6 ± 0.8 | 0 |
| Eucalyptol | 154.24 | 0.9 | 1.6 ± 0.3 | 39.4 ± 3.0 | 9 |
| Eugenol | 164.20 | 1.1 | 0.0 ± 0.5 | 51.2 ± 3.0 | 29 |
| Isomenthol | 156.26 | 0.9 | 0.0 ± 0.9 | 52.5 ± 6.0 | 20 |
| D-Limonene | 136.23 | 0.8 | 1.5 ± 0.2 | 39.5 ± 0.8 | 0 |
| Neoisocarvomenthol | 156.26 | 0.9 | 0.0 ± 0.9 | 51.7 ± 6.0 | 20 |
| L-Menthone | 154.24 | 0.9 | 0.3 ± 0.4 | 44.1 ± 3.0 | 17 |
| Menthyl acetate | 198.30 | 0.9 | 0.1 ± 0.5 | 46.6 ± 3.0 | 26 |
| à-Pinene | 136.23 | 0.9 | 3.5 ± 0.1 | 37.8 ± 0.8 | 0 |
| Terpinolene | 136.23 | 0.9 | 1.1 ± 0.2 | 40.1 ± 0.8 | 0 |
| ***Conventional fumigants*** | | | | | |
| Methyl bromide | 94.93 | 1.6 | 1589.5 ± 0.0 | 23.9 ± 0.0 | 0 |
| Phosphine | 33.99 | 1.3 | 27274.0 ± 0.1 | 14.6 ± 0.0 | 0 |

**S1 Table**

*Note:* The above parameters and values for each chemical were obtained from the chemspider site (website http://www.chemspider.com/).
